# Hand infections: a retrospective analysis

**DOI:** 10.7717/peerj.513

**Published:** 2014-09-02

**Authors:** Tolga Türker, Nicole Capdarest-Arest, Spencer T. Bertoch, Erik C. Bakken, Susan E. Hoover, Jiyao Zou

**Affiliations:** 1 Department of Orthopaedic Surgery, The University of Arizona, Tucson, AZ, USA; 2 Arizona Health Sciences Library, The University of Arizona, Tucson, AZ, USA; 3 The University of Arizona College of Medicine, Tucson, AZ, USA; 4 Sanford Health Infectious Diseases, Sioux Falls, SD, USA; 5 Division of Reconstructive and Plastic Surgery, Department of Surgery, The University of Arizona College of Medicine, Tucson, AZ, USA

**Keywords:** Antibiotics, Hand, Incision-drainage, Intravenous drug usage, Methicillin-resistant *Staphylococcus aureus* (MRSA), Infection

## Abstract

**Purpose.** Hand infections are common, usually resulting from an untreated injury. In this retrospective study, we report on hand infection cases needing surgical drainage in order to assess patient demographics, causation of infection, clinical course, and clinical management.

**Methods.** Medical records of patients presenting with hand infections, excluding post-surgical infections, treated with incision and debridement over a one-year period were reviewed. Patient demographics; past medical history; infection site(s) and causation; intervals between onset of infection, hospital admission, surgical intervention and days of hospitalization; gram stains and cultures; choice of antibiotics; complications; and outcomes were reviewed.

**Results.** Most infections were caused by laceration and the most common site of infection was the palm or dorsum of the hand. Mean length of hospitalization was 6 days. Methicillin-resistant *Staphylococcus aureus*, beta-hemolytic Streptococcus and methicillin-susceptible S*taphylococcus aureus* were the most commonly cultured microorganisms. Cephalosporins, clindamycin, amoxicillin/clavulanate, penicillin, vancomycin, and trimethoprim/sulfamethoxazole were major antibiotic choices. Amputations and contracture were the primary complications.

**Conclusions.** Surgery along with medical management were key to treatment and most soft tissue infections resolved without further complications. With prompt and appropriate care, most hand infection patients can achieve full resolution of their infection.

## Introduction

Hand infections are common occurrences, usually resulting from an injury, that when left untreated can quickly lead to tissue destruction and loss of function or permanent disability. Infections may be categorized anatomically: superficial, involving the tendon and tendon sheath, involving joint or bone, or affecting the deep spaces of the hand. Infections may be caused by different microorganisms, and, increasingly, by community-acquired methicillin-resistant *Staphylococcus aureus* (MRSA). Currently MRSA accounts for as much as 65% of *Staphylococcus aureus* isolates, complicating the course of medical treatment. In this retrospective study, we have reviewed and reported on hand infection cases needing surgical drainage at two affiliated teaching hospitals in order to assess patient demographics, causation of infection, clinical course, operative cultures including prevalence of MRSA, and clinical management.

## Materials and Methods

A University of Arizona IRB-approved retrospective review of the electronic medical records of patients presenting with hand infections, excluding post-surgical infections, treated with incision and debridement by two hand surgeons over a one-year period was conducted (IRB #12-0671-01). This was a retrospective chart review. Procedures followed were in accordance with the ethical standards of the responsible committee on human experimentation (institutional and national) and with the Helsinki Declaration of 1975, as revised in 2000 and 2008 — N/A (retrospective chart review). No identifying patient information is used in this manuscript. Patient charts were excluded for incomplete demographic information or unavailable outcome results. All patient information was de-identified between chart review and database recording. The database that was constructed from chart review included patient demographics; significant past medical history (e.g., diabetes, intravenous drug use, and immune-compromised conditions); infection site(s) and causation; intervals between onset of infection, hospital admission, surgical intervention and days of hospitalization; gram stains and cultures; choice of antibiotics; complications; and outcomes.

Infection diagnosis was classified on a 6-point scale: superficial/cellulitis, tenosynovitis, deep infection, necrotizing-type infection, bone infection, and septic arthritis. Each patient’s infection diagnosis was classified according to this scale and according to whether they had one or multiple diagnoses. All patients were treated with incision and drainage and additional debridement when required. Antibiotics were started empirically and then adjusted, if necessary, according to culture results.

## Results

One hundred twenty-three patient charts were reviewed and 94 patient charts were included. The ratio of male to female patients was 2:1 (71.3% were male, 28.7% female). Mean patient age was 42 years (SD ± 16). The mean inpatient length of stay (LOS) was 6 days (SD ± 9). Twenty-nine patients had hospital stays of 6 or more days; most of these patients had either laceration or human bite injuries, while 14% of these patients could not identify a cause of infection (Table 1). Mean days pre-hospitalization (injury to presentation at the ER) was 7 days (SD ± 9), and mean time to surgery (presentation at the ER to surgery) was 0.5 days (SD ± 0.9).

**Table 1:** Etiology of hand infections.

Cause	Total no. patients (*n* = 94)	% patients	No. patients w/MRSA culture (*n* = 28)	% patients w/MRSA culture	LOS ≥ 6 days (*n* = 29)	% Patients w/LOS ≥ 6 days
Self cut or sharp cut (laceration)	20	21%	8	28%	6	21%
Unknown	16	17%	5	18%	4	14%
Thorn	12	13%	0	0	3	10%
Human bite injury	10	11%	1	4%	5	18%
Injection (IV drug injection)	10	11%	7	25%	2	7%
Dog bite	9	10%	3	11%	3	10%
Insect bite	8	9%	2	7%	4	14%
Blunt trauma	6	6%	2	7%	1	3%
Cat bite	1	1%	0	0	0	0
Snake bite	1	1%	0	0	1	3%
Pressure injection (paint)	1	1%	0	0	0	0

Infections occurred about equally in right (46%) and left (49%) hands, and 5% of patients presented with simultaneous infection in both hands. The most common site for injury and subsequent infection was the palm and dorsal side of hand (21%), the middle finger or third web space (17%), and the index finger and second web space (16%) (Table 2).

**Table 2:** Location zone of infection.

Location zone	All patients (*n* = 94), this study		All patients (*n* = 64), (Phipps & Blanshard, 1992)		All patients (*n* = 102), (Nourbakhsh et al., 2010)		MRSA (*n* = 28), this study		Injection/IV drug use (*n* = 10), this study	
	No. pts.	% pts.	No. pts.	% pts.	No. pts.	% pts.	No. pts.	% pts.	No. pts.	% pts.
Thumb & 1st web space	13	14%	9	14%	5	5%	3	11%	2	20%
Index finger & 2nd web space	15	16%	15	23%	6	6%	3	11%	0	0
Middle finger & 3rd web space	16	17%	15	23%	5	5%	5	17%	1	10%
Ring finger & 4th web space	7	7%	7	14%	13	13%	3	11%	0	0
Small finger	8	9%	2	3%	15	15%	2	7%	0	0
Palm & dorsal side of hand	20	21%	13	20%	37	36%	9	32%	5	50%
Wrist & forearm	7	7%	n/a	n/a	12	12%	1	4%	0	0
Elbow	2	2%	n/a	n/a	n/a	n/a	0	0	0	0
Multiple zones	7	7%	n/a	n/a	9	8%	2	7%	2	20%

The most frequent infection diagnosis was deep infection (77 patients) (Table 3). Patient age, gender, insurance status, comorbidities, cause of injury to hand, location of injury, or interval between time of injury to presentation at the ER were not significant predictors of LOS. Eighty-five of 94 patients required only a single-stage surgical procedure. In 1 of these cases, the patient developed complex regional reflex dystrophy that resolved in 6 months. Three patients required 2 additional debridements that resulted in amputation. One patient with a necrotizing infection required 3 additional surgeries and, ultimately, the amputation of 1 finger. Two patients required 2 debridements and developed PIP joint contracture. Two patients required 2 debridements and subsequently recovered uneventfully. In each of the foregoing cases, patients had chronic co-morbidities (e.g., diabetes) and/or severe infection (e.g., osteomyelitis or septic arthritis). In the rest of the cases, the infection resolved without incident. The average length of time for follow-up was 7 weeks for soft tissue infections and 3–7 months for osteomyelitis or septic arthritis.

**Table 3:** Classification of infection diagnosis.

Classification/diagnosis	No. of patients (*n* = 94)	% patients	No. patients with LOS over 5 days (*n* = 29)	% patients with LOS over 5 days
Superficial or cellulitis	3	3%	1	33%
Tensynovitis	5	5%	0	0
Deep infection^a^	72	77%	18	25%
Necrotizing-type infection	1	1%	Unknown	n/a
Osteomyelitis	2	2%	2	100%
Multiple diagnoses (including septic arthritis^b^)	11	12%	8	73%

**Notes.**

a Deep infections also occurred in 5 patients with multiple diagnoses.

b Septic arthritis was always part of a multiple diagnosis (e.g., septic arthritis and tensynovitis). Three patients had septic arthritis.

Cultures were obtained from tissue excised from the infection sites at the time of surgery. The three most commonly isolated microorganisms were MRSA (28 patients), beta-hemolytic Streptococcus (22 patients), and methicillin-susceptible S*taphylococcus aureus* (MSSA) (21 patients) (Table 4). In 13 out of 94 cases, both Gram stains and cultures were negative. In 24 patients (29 organisms), Gram stains were negative but cultures were positive. This group included MRSA (5), MSSA (3), alpha-hemolytic Streptococcus (5), Enterococcus (3), beta-hemolytic Streptococcus (7), *Pasteurella* (1), *Eikenella* (1), *Morganella* (1), *Nocardia* (1), and *Klebsiella* (2). In 3 cases, Gram stains showed gram-positive cocci, but the cultures remained sterile.

**Table 4:** Cultured microorganisms.

Microorganism	No. of patients with pure culture	No. of patients with mixed culture
MRSA	20	8
Beta-hemolytic Streptococcus	11	11
MSSA	13	8
Alpha-hemolytic Streptococcus	10	2
Enterococcus	2	3
*Eikenella*	1	1
*Klebsiella*	1	1
*Morganella*	1	0
*Serratia*	1	0
*Bacillus* ^a^	0	1
Anaerobic gram negative^a^	0	1
*Neisseria* ^a^	0	1
*Nocardia* ^a^	0	1
*Pasteurella* ^a^	0	3
*Sporothrix schenckii* ^a^	0	1

**Notes.**

Some patient cultures were positive for more than one organism, as indicated.

a These microorganisms were always cultured with an additional microorganism(s) (e.g., *Pasteurella* and beta-hemolytic Streptococcus).

Medical management included 19 different antibiotics and 1 antifungal medication. The majority of these medications were started at the ER or the referring physician office; however, the majority of the antibiotics were subsequently tapered down to a cephalosporin, clindamycin, amoxicillin/clavulanate, penicillin, vancomycin, or trimethoprim/sulfamethoxazole after consulting with infectious disease specialists or empirically by the hand surgeons. Patients with MRSA (20 patients) were clinically responsive to treatment with IV and/or oral clindamycin (10), sulfamethoxazole/trimethoprim (2), vancomycin (3), or a combination thereof (5). Patients with beta-hemolytic Streptococcus (11 patients) clinically responded to a variety of antibiotics, including amoxicillin/clavulanate (2), ceftriaxone (1), clindamycin (2), sulfamethoxazole/trimethoprim (1), penicillin (2), or one or more of these drugs combined with ciprofloxacin (3). Patients with MSSA (13 patients) were often treated with polytherapy (9) primarily using drug combinations that included clindamycin, a cephalosporin or vancomycin. Antibiotics were adjusted when MSSA was identified. Patients with cultures of the other 12 microorganisms often presented either with (i) mixed culture (e.g., MSSA and *Bacillius*) that was treated with the taper regimen described above, or (ii) single culture (e.g., *Serratia*) that was then treated according to reported susceptibility.

## Discussion

In this study, we report hand infection characteristics in 94 patients who presented to the ER and were treated with surgical drainage and antibiotics. Even though hand infections can be seen in patients of every age, the average age of patients in this study group was 42 years. Given the large standard deviation of 16 years, it can be seen that this problem spans a wide variety of age groups and is not just a problem of middle age. These demographics are parallel to those of other studies previously published over several decades (Houshian, Seyedipour & Wedderkopp, 2006; Imahara & Friedrich, 2010; Nunley et al., 1980).

Brown & Young (1993) state that major metropolitan hospitals should expect 25–50 admissions annually for serious hand infection. Akdemir & Lineaweaver (2011) and Phipps & Blanshard (1992) reported fewer than 15 patients per year in each of their series. In contrast to these, our 123 patients in one year is a much higher number. Furthermore, our service treats only patients needing surgical drainage; therefore we can conjecture that the rate of hand infection in our institution and community is even higher than reported here.

Hand infections primarily occur after delayed treatment after minor trauma. Laceration, unknown causation, thorn, human bite (e.g., often “fight-bite” injuries), IV injection injury, dog bite, insect bite, blunt trauma, cat bite, snake bite, and pressure injection were the causes of infections in this study (Table 1). These causes mirror results of other published studies (Akdemir & Lineaweaver, 2011; Brown & Young, 1993; Nunley et al., 1980; Phipps & Blanshard, 1992); however we believe that the single infection caused by snake bite and the 12 infections caused by thorns in this study are most likely due to geographic location. Others have reported that 60% of the infections were due to trauma, 25–30% human bites, 10–15% drug abuse, and 5–10% animal bites (Brown & Young, 1993; Nunley et al., 1980). The majority of hand infections resulted from lacerations in Phipps and Blanshard’s study; however they reported only one hand infection that was possibly related to IV drug abuse (Phipps & Blanshard, 1992). In this study, 10 patients (11%) presented due to infection caused by IV drug use. We conjecture that this number is actually higher because patients may be reluctant to provide an accurate report about the cause of injury when it is related to IV drug use. It is possible that some of the reported “unknown” causes of injury (16%) in this study were due to IV drug use. Infection in drug users is related to either dirty needles or extravasation of the drug (Brown & Young, 1993). As many organizations have instituted clean needle exchange programs to reduce the spread of HIV, it may be interesting to investigate whether, in locales where such programs are in place, there is reduced incidence of hand infections among IV drug users.

The palm or dorsum of the hand, followed by the middle finger and third web space and then the index finger and second web space were the most frequent locations of infection (Table 2). Phipps & Blanshard (1992) reported that the most common sites for infection were in the index and middle fingers, followed by the palm/dorsum and then the thumb. Nourbakhsh et al. (2010) reported that the most common site of hand infection is at the hand (palm/dorsum) level followed by the small and ring fingers. Taken together, the palm and dorsum of the hand seem to be the areas most prone to infection (Table 2). Our data suggests that IV drug use may be contributing to the higher incidence of palm/dorsal hand infections. For patients with MRSA infections (28), most infections were also located on the palm or dorsal aspect of the hand (11), followed by the middle finger and third web space (5), and the thumb and first web space (4) (Table 2). It thus seems that MRSA does not have a predilection for a particular part of the hand.

Brown & Young (1993) reported that the most common hand infections are cellulitis and paronychia/eponychia (70%) and more severe infections such as septic arthritis and osteomyelitis occur less often (3%). Anwar, Tzafetta & Southern (2008) also reported that majority of infections were cellulitis and abscess. In our study, the majority of patients had cellulitis and deep abscess, there were no patients with paronychia, and there were 2 felon patients (Table 3). As mentioned above, in order to gain a more accurate picture of hand infection epidemiology, studies should be conducted to investigate distribution and incidence using data from a variety of care facilities.

The severity of hand infections may be classified in various ways. Brown (1978) classified hand infections as deep or superficial infections according to localization, however we believe that the infection in hand or forearm should be considered as a superficial infection if an infection involves only the epidermis and dermis. In the hand, however, since the depth of layers are thinner than those in the forearm, and once the infection involves subcutaneous tissue, we consider it as a deep infection. Previous studies report that most infections are subcutaneous as opposed to more severe infections such as osteomyelitis (Glass, 1982; Houshian, Seyedipour & Wedderkopp, 2006). Our results are consistent with these findings (Table 3). Most of our patients who presented with deep infections waited over 3 days before presenting to the ER. It is possible that in these cases, the delay in treatment caused the severity of the infection to increase.

The most frequently isolated microorganism in our study (30% of cases) was MRSA, similar to most other articles on hand infections (Anwar, Tzafetta & Southern, 2008; Brown & Young, 1993; Eaton & Butsch, 1970; McDonald et al., 2011; Nunley et al., 1980). Fowler & Ilyas’s (2013) report on 1,507 incision and drainage patients showed 53% of the cases were due to MRSA. Review of the last 20 years of publications describes increasing occurrence of MRSA along with increasing resistance to antibiotic treatment (Akdemir & Lineaweaver, 2011; Kiran, McCampbell & Angeles, 2006; LeBlanc et al., 2007; Nunley et al., 1980). Thirteen percent of our cases had negative Gram stains and cultures. These results are slightly higher than those of Nunley et al. (1980), who reported 3.5% of their patients had negative results on both Gram stain and culture and Houshian, Seyedipour & Wedderkopp (2006), who reported 11% sterile cultures. Fowler & Ilyas (2013) reported 70% sterile cultures in 1,507 patients, however 50% of their patients had paronychia, which was most likely treated with antibiotics by other physicians before surgery was performed. Negative Gram stain and culture results may be due to prior antibiotic treatment or improper specimen handling.

A wide variety of approaches to antibiotic treatment have been used and evolved over the years as antibiotic resistance and occurrence of community-acquired antibiotic-resistant infections has increased (Akdemir & Lineaweaver, 2011; Brown & Young, 1993; Brown, 1978; Eaton & Butsch, 1970; Houshian, Seyedipour & Wedderkopp, 2006; Imahara & Friedrich, 2010; McDonald et al., 2011; Nicholls, 1973; Nunley et al., 1980; Weinzweig & Gonzalez, 2002). Even though the choice of antibiotics is evolving from those used in the past due to changes in microorganisms and developing antibiotic resistance, the treatment principles set forth by Brown & Young (1993) of 3–5 days of IV antibiotic treatment followed by 7–10 days of oral antibiotic treatment remains a valid treatment plan for hand infection patients. Our study supports these treatment principles as most of our patients received IV antibiotics during inpatient treatment and then discharged with oral antibiotics.

Another important issue in hand infections is delay in seeking treatment and/or delayed surgical drainage. Glass reported that delay in treatment causes slower resolution; for example, if the delay is more than 2.5 days, about 70% of those patients showed delayed recovery (Glass, 1982). In this study, the time interval between presentation to the ER and surgery was usually within 24 h (95% underwent surgical procedure within 24 h of presenting to the ER).

Surgical treatment is an important component of clinical management. Some have reported a high need for multiple surgeries in managing hand infections (Imahara & Friedrich, 2010). This may be due to severity of infection and/or limited surgical approach. A detailed, comprehensive initial surgical approach may minimize the need for multiple surgeries. In our series, patients only required multiple surgeries in cases of osteomyelitis, septic arthritis or necrotizing infection. Intraoperative care was comprehensive, using copious Dakin’s solution, hydrogen peroxide, sterile water, and bacitracin in normal saline to irrigate the infected area. Postoperatively, we also use Dakin’s solution for immediate soaking of the open wound 1–2 times per day for 10 min for 3 days. Carter and Mersheimer’s study suggests that after the pus is removed from an infected hand, an antibiotic drip should be inserted to the infected area and advises against soaking because tissue damage by maceration or burns may occur with warm water soaking (Carter & Mersheimer, 1970). Another difference in our treatment approach involves the use of soft dressing instead of splinting. Although traditionally, post-operative splinting may be suggested for hand infection treatment (Kiran, McCampbell & Angeles, 2006), we do not use postoperative splinting and, instead, we apply soft dressing and start early range of motion in order to decrease stiffness. All patients tolerated this regimen well and we believe it is helpful in promoting enhanced function.

Our study has some limitations. We were not able to obtain comprehensive data for all treated patients. We had 123 patients who underwent surgical drainage but we could only retrieve necessary data for 94 patients due to insufficient data in the electronic health record. Additionally, we were unable to provide detailed comparison with older studies regarding specific pathogens due to limitations in microbiology results. Co-morbidities have not been discussed here since, perhaps due to the limited sample size, we did not find any significant differences in outcomes in patients with co-morbidites (e.g., diabetes, immunosuppression). Finally, part of the nature of how patients progress through the health care system as they seek treatment for hand infection often involves multiple care providers and multiple facilities. This makes it difficult to accurately capture the entire course of antibiotic treatment obtained by the patients.

In conclusion, our review demonstrated that, with prompt and appropriate care, most soft tissue hand infection patients can achieve full resolution of their infections. Treating infections promptly with surgical incision and drainage using a large incision with copious irrigation and then using a regimen of soaking and use of soft dressing with early range of motion exercises are key to achieving good outcomes and avoiding multiple surgical procedures. In conjunction with surgical treatment, judicious antibiotic treatment is also important. After cultures are obtained, antibiotic treatment should be tailored more specifically to the organisms. Consultation with an infectious diseases specialist can sometimes be helpful in choosing the most appropriate regimen. Lastly, as it seems that the incidence hand infections caused by IV drug injection is increasing, we recommend increased patient education for IV drug users regarding the risk of hand infection, the importance of using clean needles and good hand hygiene, and substance abuse treatment programs.
